# Validity of estimated prevalence of decreased kidney function and renal replacement therapy from primary care electronic health records compared with national survey and registry data in the United Kingdom

**DOI:** 10.1093/ndt/gfw318

**Published:** 2017-02-11

**Authors:** Masao Iwagami, Laurie A. Tomlinson, Kathryn E. Mansfield, Anna Casula, Fergus J. Caskey, Grant Aitken, Simon D.S. Fraser, Paul J. Roderick, Dorothea Nitsch

**Affiliations:** 1 Department of Non-Communicable Disease Epidemiology, London School of Hygiene and Tropical Medicine, London, UK; 2 UK Renal Registry Bristol, UK; 3 Geography & Environment, Faculty of Social and Human Sciences, University of Southampton, Southampton, UK; 4 Academic Unit of Primary Care and Population Sciences, Faculty of Medicine, University of Southampton, Southampton, UK

**Keywords:** chronic kidney disease, epidemiology, primary care, renal replacement therapy, validity

## Abstract

**Background:**

Anonymous primary care records are an important resource for observational studies. However, their external validity is unknown in identifying the prevalence of decreased kidney function and renal replacement therapy (RRT). We thus compared the prevalence of decreased kidney function and RRT in the Clinical Practice Research Datalink (CPRD) with a nationally representative survey and national registry.

**Methods:**

Among all people ≥25 years of age registered in the CPRD for ≥1 year on 31 March 2014, we identified patients with an estimated glomerular filtration rate (eGFR) <60 mL/min/1.73 m^2^, according to their most recent serum creatinine in the past 5 years using the Chronic Kidney Disease Epidemiology Collaboration equation and patients with recorded diagnoses of RRT. Denominators were the entire population in each age–sex band irrespective of creatinine measurement. The prevalence of eGFR <60 mL/min/1.73 m^2^ was compared with that in the Health Survey for England (HSE) 2009/2010 and the prevalence of RRT was compared with that in the UK Renal Registry (UKRR) 2014.

**Results:**

We analysed 2 761 755 people in CPRD [mean age 53 (SD 17) years, men 49%], of whom 189 581 (6.86%) had an eGFR <60 mL/min/1.73 m^2^ and 3293 (0.12%) were on RRT. The prevalence of eGFR <60 mL/min/1.73 m^2^ in CPRD was similar to that in the HSE and the prevalence of RRT was close to that in the UKRR across all age groups in men and women, although the small number of younger patients with an eGFR <60 mL/min/1.73 m^2^ in the HSE might have hampered precise comparison.

**Conclusions:**

UK primary care data have good external validity for the prevalence of decreased kidney function and RRT.

## BACKGROUND

Chronic kidney disease (CKD) is a major public health problem that increases in prevalence with age and is associated with increased morbidity and mortality [1–3]. The number of people with end-stage renal disease (ESRD) requiring renal replacement therapy (RRT) has been increasing worldwide and is predicted to double by 2030 [4]. Appropriate identification of CKD is thus important for early intervention, including prevention of both CKD progression and cardiovascular diseases [5]. At the population level, accurate estimation of CKD prevalence is essential to assess the burden of CKD in the community and to evaluate the effectiveness of population approaches for CKD [6]. However, potential methodological difficulties may make it problematic to determine the community prevalence of CKD [7, 8]. For example, people who have kidney function measured routinely by serum creatinine may not represent the general population and serum creatinine assays may not be uniformly standardized.

Data derived from routine patient care, such as the anonymous primary care records held in the UK Clinical Practice Research Datalink (CPRD) [9], are an important resource for observational studies [10]. Because CRPD broadly represents the UK population in terms of demographics [11], it can be a useful source to estimate a disease prevalence in the UK. However, using routine electronic records to investigate renal disease is only possible if the general practitioners (GPs) appropriately test, identify and record everyone in the population who has kidney disease. Reliable measures of renal disease in electronic health records would allow a more robust use of primary care data to investigate renal disease epidemiology; for example, researchers would be able to investigate the association between kidney diseases and other comorbidities or medications recorded in primary care data. To date, a number of definitions for diseases or specific conditions have been validated in the CPRD at the individual or population level [12, 13]. However, to our knowledge, there has been no external validation study for the prevalence of decreased kidney function and RRT in the CPRD. The best available methods to identify CKD and RRT in CPRD are to use serum creatinine records measured by GPs and recorded diagnoses of RRT in the CPRD, respectively, yet the validity or appropriateness of these methods are unknown.

The Health Survey for England (HSE), a nationally representative survey of health condition, included measurement of kidney function in 2009 and 2010 [14]. Every consenting participant had kidney function measured, giving representative statistics for the prevalence of decreased kidney function in the general population. Meanwhile, the UK Renal Registry (UKRR), which records information regarding all people on RRT in the UK, provides annual reports of the prevalence of RRT [15]. Referring to these two nationally representative sources of data, we aimed to evaluate the external validity of the prevalence of decreased kidney function and RRT in the CPRD.

## MATERIALS AND METHODS

### Details of the CPRD and study population

In the UK, the primary care system acts as a gatekeeper to health care—patients need to be registered with a primary care doctor to access National Health Service (NHS) non-emergency care. Health care is free at the point of access. Primary care practices have used computerized electronic health records since the early 1990s. There are only a limited number of suppliers of GP electronic health record software. The CPRD uses data from VISION software system (In Practice Systems, London, UK) and has evolved as an observational data and interventional research service provided by the NHS. Currently >650 GP practices contribute data meeting quality control standards to the CPRD, covering and representing nearly 7% of the UK population [11]. Previous studies have suggested that the distribution of age, sex, ethnicity, practice location deprivation, and other health indicators such as smoking and morbidities are similar to that of external UK-based sources [11, 16–19]. The database includes patient demographics, coded diagnoses and outpatient laboratory test results. The Secretary of State waived informed consent for CPRD data because data are anonymized and there is an overall benefit for research. Ethical approval for this study was obtained from the Independent Scientific Advisory Committee, which oversees research on CPRD data (protocol no. 16_055), as well as the London School of Hygiene and Tropical Medicine Ethics Committee (reference: 9196).

The study population was all people ≥25 years of age who were alive and registered in the CPRD for at least 1 year on 31 March 2014. The choice of age 25 years as a lower limit was made for the best comparability between the CPRD and HSE or UKRR: the HSE and UKRR collected data of people <25 years of age differently (the HSE grouped people 16–24 years of age, while the UKRR grouped people 18–24 years of age). One-year registration was considered necessary for GPs to record a history of RRT for newly registered patients or to test their kidney function if they had a key CKD risk factor such as diabetes [5].

### Details of external data

For the prevalence of decreased kidney function, we compared the data from the CPRD with those from the HSE 2009 and 2010 (combined) [14]. Briefly, the HSE 2009/2010 included a cross-sectional study of kidney disease among people selected using a multistage stratified random probability sampling method. Blood samples were taken from nearly 6000 consenting participants, accounting for 77% for men and 73% for women among all the HSE participants. Data were weighted for non-response to reduce response bias. Creatinine was measured by an internationally standardized enzymatic method, which is traceable to isotope dilution mass spectrometry (IDMS) [20]. Estimated glomerular filtration rate (eGFR) was calculated from the serum creatinine value using the Modification of Diet in Renal Disease Study equation in the original HSE report [14], whereas a *post hoc* analysis was conducted using the Chronic Kidney Disease Epidemiology Collaboration (CKD-EPI) equation [21]. The prevalence of people with a single eGFR <60 mL/min/1.73 m^2^ was reported according to age (every 10 years) and sex.

For RRT prevalence, we referred to the data from the UKRR 2014 [15]. The UKRR 2014 collected data from all 71 renal centres in the UK. The prevalence of RRT in 2013 was estimated by dividing the number of patients on RRT by the 2013 UK population, according to age (every 10 years), sex and RRT modality: haemodialysis, peritoneal dialysis or kidney transplantation.

### Definition of decreased kidney function and RRT in the CPRD

We identified patients with an eGFR <60 mL/min/1.73 m^2^ according to their most recent single serum creatinine measured by a GP in the past 5 years (i.e. the period between 1 April 2009 and 31 March 2014) using the CKD-EPI equation [22]. We used a single eGFR to define decreased kidney function in the main analysis because the HSE (reference data in this study), as well as previous large epidemiological studies [23, 24], have used this definition. For the main analysis, we made the following assumptions: (i) all the UK laboratories reported IDMS-traceable creatinine; (ii) people with a missing record of ethnicity in the CPRD had non-black ethnicity and (iii) people without any creatinine measurement for the past 5 years did not have decreased kidney function.

We identified patients on RRT based on the diagnoses recorded in the CPRD anytime from the date of their registration to 31 March 2014. The list of diagnosis codes (Read codes) indicative of RRT was determined by using a recommended strategy [25] and agreed upon among the authors (Supplementary data, Table S1). In addition, in order to examine the validity of diagnoses of different RRT modality in CPRD, we classified patients with RRT into those with haemodialysis, peritoneal dialysis or kidney transplantation. We used the most recent recorded diagnosis, as this is the best available approach to estimate the prevalence of the current RRT modality in CPRD.

### Data analysis

We calculated the prevalence [95% confidence interval (CI)] of eGFR <60 mL/min/1.73 m^2^ according to age (every 10 years) and sex in the CPRD and HSE, respectively, using the CKD-EPI equation. Denominators in the CPRD were the entire population in each age–sex band irrespective of creatinine measurement in the past 5 years. Patients ≥75 years of age were grouped in the CPRD to be consistent with the HSE. We calculated the difference (95% CI) in the prevalence of eGFR <60 mL/min/1.73 m^2^ between the CPRD and HSE. We also reported the proportion of patients with at least one creatinine measurement for the past 5 years in the CPRD.

Similarly, we calculated the prevalence of RRT in the CPRD and UKRR, respectively, and then the difference between the CPRD and UKRR, in 10-year age bands by sex. We also reported results by RRT modality.

All statistical analyses were conducted using Stata 14 software (StataCorp, College Station, TX, USA).

### Sensitivity analyses

We repeated our analyses using a number of alternative eGFR definitions and restricted study populations in order to determine the impact of the definition for decreased kidney function that we used. We defined decreased kidney function as follows: (i) we assumed that all the UK laboratories reported non-IDMS-traceable creatinine, and therefore multiplied the recorded creatinine value by 0.95 to use the CKD-EPI equation for IDMS-traceable creatinine [26]; (ii) we conducted a complete case analysis for ethnicity (restricting the analysis to people with recorded ethnicity in the CPRD); (iii) we used the participants' most recent creatinine in the past 2 years, instead of 5 years; (iv) we restricted the region to England, by excluding data from Scotland, Wales and Northern Ireland; (v) we additionally required a measure of chronicity to define decreased kidney function [27]: two or more eGFR results <60 mL/min/1.73 m^2^ needed to be recorded consecutively ≥3 months apart in the past 5 years; and (vi) we conducted a complete case analysis for creatinine by restricting the analysis to people with at least one creatinine measurement in the past 5 years.

We also compared the prevalence of eGFR <45 mL/min/1.73 m^2^ (calculated from the most recent creatinine in the past 5 years) between the CPRD and HSE, which may be a more robust indicator of decreased kidney function with prognostic implications [28, 29].

## RESULTS

From 685 GP practices, we identified 2 761 755 people [mean age 53 (SD 17) years, men 49%] who were alive and registered in the CPRD for ≥1 year on 31 March 2014. Their age–sex distribution was broadly similar to that of the UK Census 2013 (Supplementary data, Table S2). Of those identified, 189 581 patients (6.86%) had an eGFR <60 mL/min/1.73 m^2^ and 3293 patients (0.12%) were on RRT.

**FIGURE 1:**
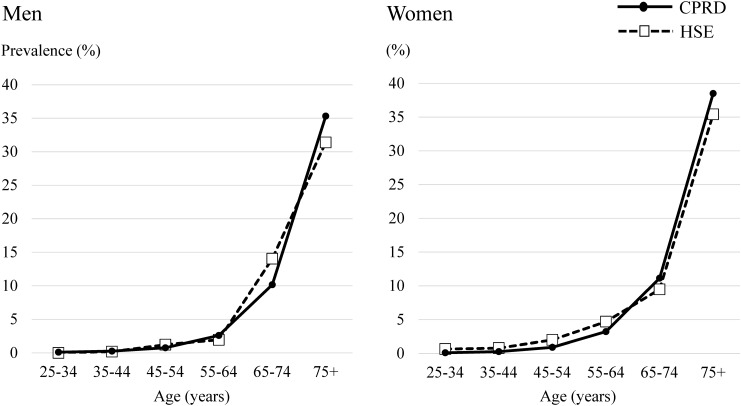
Prevalence of eGFR <60 mL/min/1.73 m^2^ in the CPRD and HSE.

The prevalence of eGFR <60 mL/min/1.73 m^2^ increased steeply with age (Table 1 and Figure 1). There was no evidence that the prevalence of eGFR <60 mL/min/1.73 m^2^ in the CPRD was different from that in the HSE across age groups, both in men and women, except for the group of men 25–34 years of age, in which no one had an eGFR <60 mL/min/1.73 m^2^ in the HSE. The proportion of people who had a recorded measurement of creatinine increased with age, with 26% of men and 46% of women 25–34 years of age with tests in the past 5 years, up to 92% (both men and women) among people 75 years of age.

**Table 1.** Prevalence of eGFR <60 mL/min/1.73 m^2^ in the CPRD and HSE

	Age group (years)					
	25–34	35–44	45–54	55–64	65–74	≥75
Men						
Prevalence of eGFR <60 mL/min/1.73 m^2^ in CPRD, % (95% CI)	0.11 (0.10–0.12)	0.27 (0.25–0.29)	0.76 (0.73–0.79)	2.59 (2.53–2.66)	10.16 (10.02–10.29)	35.32 (35.07–35.57)
Prevalence of eGFR <60 mL/min/1.73 m^2^ in HSE, % (95% CI)	0	0.19 (0–1.06)	1.22 (0.45–2.63)	1.94 (0.84–3.78)	14.04 (10.27–18.56)	31.39 (25.36–37.92)
Difference (prevalence in CPRD−HSE), % (95% CI)	0.11 (0.09–0.12)	0.08 (−0.30–0.45)	−0.46 (−1.43–0.51)	0.65 (−0.68–1.99)	−3.88 (−7.87–0.10)	3.93 (−2.17–10.03)
Proportion of patients with serum creatinine measurement in past 5 years in CPRD, % (numerator/denominator)	25.85 (61 339/237 284)	38.47 (98 759/256 739)	55.61 (163 001/293 104)	72.44 (167 841/231 695)	86.15 (163 933/190 292)	92.29 (132 103/143 144)
Women						
Prevalence of eGFR <60 mL/min/1.73 m^2^ in CPRD, % (95% CI)	0.10 (0.09–0.12)	0.27 (0.25–0.29)	0.90 (0.87–0.94)	3.22 (3.15–3.29)	11.13 (10.99–11.27)	38.50 (38.29–38.72)
Prevalence of eGFR <60 mL/min/1.73 m^2^ in HSE, % (95% CI)	0.65 (0.13–1.89)	0.78 (0.21–1.97)	2.00 (0.96–3.64)	4.70 (2.93–7.09)	9.48 (6.53–13.19)	35.41 (30.04–41.06)
Difference (prevalence in CPRD−HSE), % (95% CI)	−0.55 (−1.29–0.19)	−0.51 (−1.27–0.25)	−1.10 (−2.32–0.13)	−1.48 (−3.44–0.48)	1.65 (−1.53–4.83)	3.09 (−2.28–8.47)
Proportion of patients with serum creatinine measurement in past 5 years in CPRD, % (numerator/denominator)	46.22 (108 767/235 341)	55.30 (139 977/253 145)	67.35 (192 872/286 386)	75.27 (174 268/231 517)	84.45 (171 620/203 227)	91.88 (183 655/199 881)

**FIGURE 2:**
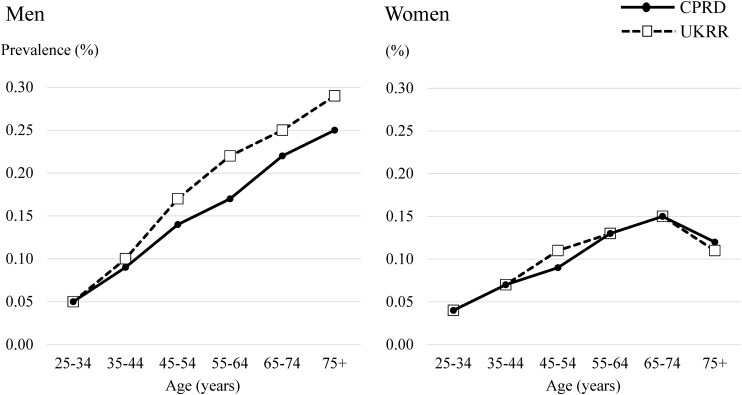
Prevalence of RRT in the CPRD and the UKRR

The prevalence of RRT gradually increased according to age (Table 2 and Figure 2). The difference between the CRPD and the UKRR was small across all age groups, both in men and women. Table 3 shows the subgroup analysis by RRT modality. The prevalence of patients with haemodialysis in the CPRD was slightly lower than that in the UKRR across all age groups, while the prevalence of those with peritoneal dialysis and kidney transplantation in the CPRD were similar to or slightly higher than those in the UKRR.

**Table 2.** Prevalence of RRT in the CPRD and UKRR

	Age group (years)					
	25–34	35–44	45–54	55–64	65–74	≥75
Men						
Prevalence of RRT in CPRD, % (95% CI)	0.05 (0.04–0.06)	0.09 (0.08–0.10)	0.14 (0.12–0.15)	0.17 (0.16–0.19)	0.22 (0.20–0.24)	0.25 (0.23–0.28)
Prevalence of RRT in UKRR, % (95% CI)	0.05 (0.05–0.06)	0.10 (0.10–0.10)	0.17 (0.16–0.17)	0.22 (0.21–0.22)	0.25 (0.25–0.26)	0.29 (0.28–0.29)
Difference (prevalence in CPRD−UKRR), % (95% CI)	0 (−0.01–0.01)	−0.01 (−0.02–0)	−0.03 (−0.05–−0.02)	−0.05 (−0.06–−0.03)	−0.03 (−0.05–−0.01)	−0.03 (−0.06–0)
Women						
Prevalence of RRT in CPRD, % (95% CI)	0.04 (0.03–0.05)	0.07 (0.06–0.08)	0.09 (0.08–0.11)	0.13 (0.11–0.14)	0.15 (0.13–0.17)	0.12 (0.10–0.13)
Prevalence of RRT in UKRR, % (95% CI)	0.04 (0.03–0.04)	0.07 (0.06–0.07)	0.11 (0.10–0.11)	0.13 (0.13–0.14)	0.15 (0.15–0.16)	0.11 (0.11–0.12)
Difference (prevalence in CPRD−UKRR), % (95% CI)	0 (−0.01–0.01)	0 (−0.01–0.01)	−0.01 (−0.03–0)	−0.01 (−0.02–0.01)	0 (−0.02–0.02)	0 (−0.01–0.02)

**Table 3.** Prevalence of RRT by modality in the CPRD and UKRR

	Age group (years)					
	25–34	35–44	45–54	55–64	65–74	≥75
Clinical Practice Research Datalink						
Denominator, *N*	472 625	509 884	579 490	463 212	393 519	343 025
Number of patients with haemodialysis, *n* (%)	39 (0.08)	84 (0.16)	144 (0.25)	202 (0.44)	257 (0.65)	378 (1.10)
Number of patients with peritoneal dialysis, *n* (%)	27 (0.06)	15 (0.03)	48 (0.08)	37 (0.08)	67 (0.17)	67 (0.20)
Number of patients with kidney transplantation, *n* (%)	141 (0.30)	299 (0.59)	480 (0.83)	455 (0.98)	399 (1.01)	154 (0.45)
UK Renal Registry						
Denominator, *N*	8 676 837	8 463 148	9 030 893	7 297 460	6 030 602	5 101 203
Number of patients with haemodialysis, *n* (%)	887 (0.10)	1677 (0.20)	3513 (0.39)	4560 (0.62)	5939 (0.98)	7324 (1.44)
Number of patients with peritoneal dialysis, *n* (%)	180 (0.02)	321 (0.04)	585 (0.06)	740 (0.10)	918 (0.15)	830 (0.16)
Number of patients with kidney transplantation, *n* (%)	2836 (0.33)	5047 (0.60)	8361 (0.93)	7538 (1.03)	5224 (0.87)	1269 (0.25)

Table 4 shows the results of sensitivity analyses. By assuming all creatinine results were non-IDMS traceable, the prevalence of eGFR <60 mL/min/1.73 m^2^ in the CPRD decreased predominantly among older people, and overall prevalence decreased from 6.86 to 5.35%. Restricting to people with recorded ethnicity in the CPRD, using a serum creatinine value in the past 2 years and restricting to English data produced similar results to the main analysis. By defining decreased kidney function including a measure of chronicity, the prevalence decreased slightly in each age group, and overall prevalence decreased from 6.86 to 6.27%. Finally, in a complete case analysis (using as the denominator only those with serum creatinine tests) the prevalence of eGFR <60 mL/min/1.73 m^2^ increased substantially compared to that in the main analysis.

**Table 4.** Prevalence of eGFR <60 mL/min/1.73 m^2^ in the CPRD: results of main analysis and sensitivity analyses

	Age group (years)						Total
	25–34	35–44	45–54	55–64	65–74	≥75	
Main analysis, % (numerator/denominator)	0.11 (497/472 625)	0.27 (1360/509 884)	0.83 (4800/579 490)	2.90 (13 455/463 212)	10.66 (41 949/393 519)	37.18 (127 520/343 025)	6.86 (189 581/2 761 755)
Sensitivity analyses, % (numerator/denominator)							
(i) Assuming all creatinine results non-IDMS traceable	0.09 (429/472 625)	0.21 (1066/509 884)	0.56 (3246/579 490)	1.86 (8612/463 212)	7.45 (29 304/393 519)	30.66 (105 171/343 025)	5.35 (147 828/2 761 755)
(ii) Complete case analysis for ethnicity	0.11 (332/295 942)	0.27 (815/299 641)	0.93 (2710/292 837)	3.24 (7313/225 881)	11.26 (22 496/199 805)	38.25 (64 923/169 738)	6.64 (98 589/1 483 844)
(iii) Using creatinine records in past 2 years	0.09 (439/472 625)	0.24 (1202/509 884)	0.73 (4202/579 490)	2.59 (11 993/463 212)	9.93 (39 081/393 519)	35.08 (120 323/343 025)	6.42 (177 240/2 761 755)
(iv) Restricting region to England	0.11 (368/346 641)	0.26 (999/377 675)	0.85 (3596/423 030)	3.00 (9935/331 404)	10.71 (30 302/282 983)	37.22 (93 887/252 246)	6.91 (139 087/2 013 979)
(v) Using CKD criteria including chronicity^a^	0.07 (353/472 625)	0.19 (977/509 884)	0.56 (3234/579 490)	2.19 (10 156/463 212)	9.34 (36 770/393 519)	35.44 (121 564/343 025)	6.27 (173 054/2 761 755)
(vi) Complete case analysis for creatinine	0.29 (497/170 148)	0.57 (1360/238 786)	1.35 (4800/355 929)	3.93 (13 455/342 151)	12.50 (41 949/335 581)	40.38 (127 520/315 777)	10.78 (189 581/1 758 372)

^a^eGFR rate <60 mL/min/1.73 m^2^ twice consecutively for ≥3 months in the past 5 years.

The overall prevalence of eGFR <45 mL/min/1.73 m^2^ was 2.33% (64 425/2 761 755) in the CPRD. The number of people with an eGFR <45 mL/min/1.73 m^2^ was small and CIs of the prevalence estimates were large in the HSE (Table 5). The proportion of people with an eGFR <45 mL/min/1.73 m^2^ in the age group ≥75 years in the CPRD was significantly higher than that of the HSE, both in men and women.

**Table 5.** Prevalence of eGFR <45 mL/min/1.73 m^2^ in the CPRD and HSE

	Age group (years)					
	25–34	35–44	45–54	55–64	65–74	≥75
Men						
Prevalence of eGFR <45 mL/min/1.73 m^2^ in CPRD, % (95% CI)	0.08 (0.07–0.09)	0.16 (0.14–0.17)	0.30 (0.28–0.32)	0.66 (0.63–0.70)	2.36 (2.30–2.43)	13.49 (13.32–13.67)
Prevalence of eGFR <45 mL/min/1.73 m^2^ in HSE, % (95% CI)	0	0	0.41 (0.05–1.46)	0	4.11 (2.14–7.07)	8.97 (5.56–13.51)
Difference (prevalence in CPRD−HSE), % (95% CI)	0.08 (0.07–0.09)	0.16 (0.14–0.17)	−0.11 (−0.67–0.45)	0.66 (0.63–0.70)	−1.75 (−4.02–0.53)	4.53 (0.77–8.28)
Women						
Prevalence of eGFR <45 mL/min/1.73 m^2^ in CPRD, % (95% CI)	0.06 (0.05–0.07)	0.12 (0.11–0.14)	0.24 (0.23–0.26)	0.60 (0.57–0.63)	2.36 (2.29–2.43)	15.14 (14.99–15.30)
Prevalence of eGFR <45 mL/min/1.73 m^2^ in HSE, % (95% CI)	0	0	0.40 (0.05–1.43)	0.89 (0.24–2.28)	2.75 (1.27–5.16)	10.82 (7.57–14.86)
Difference (prevalence in CPRD−HSE), % (95% CI)	0.06 (0.05–0.07)	0.12 (0.11–0.14)	−0.16 (−0.71–0.40)	−0.29 (−1.17–0.58)	−0.39 (−2.17–1.38)	4.32 (0.83–7.81)

## DISCUSSION

In this study, we examined the external validity of the prevalence of decreased kidney function (based on serum creatinine measured by GPs) and RRT (based on recorded diagnoses) in the CPRD by comparing them with results from two nationally representative sources (the HSE and UKRR). Across all ages for men and women the prevalence of eGFR <60 mL/min/1.73 m^2^ in the CPRD was similar to that in the HSE, although the small number of younger patients with an eGFR <60 mL/min/1.73 m^2^ in the HSE might have hampered precise comparison. The prevalence of RRT in the CPRD was broadly similar to that obtained from the UKRR, although there were differences in the RRT modality-specific prevalence between the CPRD and UKRR.

Routinely collected primary care data can be a useful resource for epidemiological studies, particularly in the UK, where >98% of citizens are registered with NHS GPs [11]. Although the prevalence or incidence of various diseases in the CPRD have good comparability with other UK-based data sources [12, 13], the external validity of the prevalence of decreased kidney function and RRT has not been studied. Concerns specific to kidney diseases include that GPs do not test every registered patient's kidney function, which could lead to underestimation of the true prevalence of decreased kidney function. In our study, the proportion of people with creatinine measurement was small among young and middle-aged people, especially men. However, using the entire practice population as a denominator, the prevalence of eGFR <60 mL/min/1.73 m^2^ in the CPRD was close to that in the HSE across all age groups, both in men and women. A possible explanation would be that, in line with the current National Institute for Health and Care Excellence (NICE) guidance for CKD [5], GPs are efficiently testing kidney function for people with CKD risk factors, including hypertension, diabetes, cardiovascular diseases and hereditary kidney disease (e.g. autosomal dominant polycystic kidney disease). In addition, the Quality and Outcome Framework (QOF) incentivizes GPs to register and manage patients with CKD [30]. Since the launch of the QOF for CKD in 2006/7, the identification and management of patients with CKD have been improving in the UK [31], although there are delays in coding patients with CKD in the system [32]. In older age groups, very high proportions had undergone testing of kidney function, and it is likely that those not tested are healthier, with a lower risk of CKD.

In sensitivity analyses, we examined to what extent the prevalence estimates for decreased kidney function changed under different assumptions related to uncertainties in the CPRD. First, the estimation changed considerably with the assumption of whether the UK laboratories reported creatinines traceable to IDMS or not. We expect that most of the UK laboratories reported IDMS-traceable creatinines during the study period, yet if a few laboratories reported non-IDMS-traceable creatinines, the true prevalence of eGFR <60 mL/min/1.73 m^2^ in the CPRD would become lower than our estimation in the main analysis. Standardization of serum creatinine assays is thus important in studies regarding CKD epidemiology. Second, the assumption of non-black ethnicity for people with missing ethnicity data in the CPRD affected the prevalence estimates only slightly. This is probably because the proportion of people with black ethnicity is small in the UK, at ∼3% [18]. Third, using creatinine records for the past 2 instead of 5 years made little change to prevalence estimates for decreased kidney function. This may relate to recommendations for regular testing in line with the QOF and the current NICE guidance for CKD [5]. Fourth, in the CPRD the prevalence of eGFR <60 mL/min/1.73 m^2^ in England was similar to that in the whole UK, ensuring comparability between the HSE and CPRD in our study. Fifth, the prevalence estimates slightly decreased by using the CKD criteria including chronicity. This may suggest that some patients with a single eGFR <60 mL/min/1.73 m^2^ had transient kidney dysfunction, probably because serum creatinine was measured at the time of acute illness when they may have developed acute kidney injury. Finally, the prevalence of decreased kidney function was likely to be overestimated by restricting the denominator to only people with creatinine measurement. This suggests that GPs selectively test people at high risk of CKD, especially among younger people.

The prevalence of RRT was also similar between the CPRD and UKRR across all age groups in men and women. Patients receiving RRT are in frequent contact with kidney units, so GPs do not provide comprehensive routine care for these individuals. However, patients on RRT remain registered with their GPs and therefore we would anticipate that GPs update patient records to reflect commencement of RRT. Our results suggest that the estimated prevalence of RRT based on recorded diagnoses in the CPRD was broadly valid when compared against comprehensive UKRR. However, using the most recent diagnosis indicating RRT modality, the prevalence of haemodialysis was underestimated in the CPRD, while those of peritoneal dialysis and kidney transplantation were similar, or somewhat overestimated, especially among older people. This may be because patients with peritoneal dialysis and kidney transplantation are often healthier and have more regular contact with their GPs compared with those on haemodialysis. In addition, for patients with a change in their RRT modality (e.g. from peritoneal dialysis to haemodialysis) there may be a delay in updating the modality in the GP record. Therefore, some patients currently on haemodialysis might be misclassified into the group of peritoneal dialysis or kidney transplantation because their previous diagnoses (i.e. peritoneal dialysis or kidney transplantation) are not yet updated. Another possibility is that patients commencing haemodialysis died before this was recorded in the CPRD, given the high early mortality rates of these patients [33].

There are several limitations to our study. First, this is a cross-sectional study examining the validity of prevalence of decreased kidney function and RRT. Our results do not ensure that UK primary care data are reliable for identifying the incidence of CKD and RRT. Second, our comparison of data between the CPRD and HSE or UKRR was only at the population rather than the individual level. Our analyses did not allow us to calculate the sensitivity or specificity of RRT diagnoses. In the absence of linked data, it is possible that there was a similar extent of misclassification between cases and non-cases, resulting in an overall agreement of the prevalence estimates in the CPRD with those in the HSE and UKRR. Third, the prevalence of decreased kidney function in the HSE was the best available estimate, but not a perfect reference standard. The survey did not include people who were temporarily hospitalized for acute illness or were in residential care [14]. In addition, people with poor health might be reluctant to give a blood sample, and the existing adjustment for non-response in the HSE may not have fully dealt with this bias. This may explain the finding in our sensitivity analysis that the proportion of people with an eGFR <45 mL/min/1.73 m^2^ in the oldest age group in the CPRD was significantly higher than that of the HSE. Blood sampling was conducted on only one occasion in the HSE. Accordingly, we defined decreased kidney function in the CPRD using one serum creatinine measurement in our main analysis. However, some patients might have had their kidney function checked as a result of acute illness, and therefore their decreased kidney function might have been transient. Previous research has shown that creatinine fluctuation can affect the CKD prevalence estimates in routinely collected data [34], although the influence was not large in our study. At ∼6000, the sample size in the HSE was not small, yet the relatively wide 95% CIs for the prevalence estimates in each age–sex group hampered more precise comparisons. In particular, the number of patients with an eGFR <60 mL/min/1.73 m^2^ was small among younger age groups. We could not compare the prevalence of more severe kidney dysfunction, because patients with an eGFR <30 mL/min/1.73 m^2^ were rare, even among older people in the HSE [14]. Meanwhile, testing of albuminuria is known to be incomplete in UK primary care electronic health records [32], which prevented us from comparing the prevalence of albuminuria, or CKD stages 1 and 2, between the CPRD and HSE. Because albuminuria is an important prognostic factor in people with and without low eGFR [35], the unknown validity of albuminuria in UK primary care remains an obstacle to the study of CKD using the CPRD. Finally, our findings may not be generalizable to other GP practices in the UK if GP practices contributing to the CPRD were more likely to measure kidney function and record the diagnoses of RRT. Generalizability to primary care electronic health records in other European countries is also uncertain, because the frequency of practices such as blood testing, chronic disease monitoring, recording of diagnoses, incentives and access to public primary care clinics differ.

In the era of a rising global prevalence of ESRD [4], high-quality epidemiological research on kidney diseases is becoming more important. Routinely collected electronic health record data would play an important role for kidney research, because most patients with CKD are diagnosed and managed in primary care. Accurate identification of CKD and RRT in the CPRD would allow investigation of the association between kidney diseases and other comorbidities or medications. It is also possible to investigate equity of care (e.g. referral to nephrologists), given that the database is less biased for ascertaining advanced CKD than population surveys and disease registries. In this study, we demonstrated that identifying the prevalence of CKD and RRT is valid at the population level in the CPRD. Although further validation of individual-level data is needed, our findings support the use of UK primary care data for research into kidney disease.

## CONCLUSIONS

We examined the external validity of the prevalence of decreased kidney function and RRT in the CPRD. The prevalence of eGFR <60 mL/min/1.73 m^2^ in the CPRD was similar to that in a national sampling survey (HSE 2009/2010), and the prevalence of RRT in the CPRD was close to that obtained from a national disease registry (UKRR 2014) across all age groups, in both men and women. These findings suggest that UK primary care data can be used to identify the prevalence of decreased kidney function and RRT in future studies.

## SUPPLEMENTARY DATA

Supplementary data are available online at http://ndt.oxfordjournals.org.

## AUTHORS' CONTRIBUTIONS

M.I., L.T. and D.N. planned the study. M.I. carried out the data extraction from the Clinical Practice Research Datalink, cleaning and analysis, and drafted the manuscript. K.M. supported the data analysis and drafted the manuscript. A.C. and F.C. managed the data from the UK Renal Registry 2014. G.A., S.F. and P.R. managed the data from the Health Survey for England 2009 and 2010. All authors contributed substantially to interpretation of the results and the writing of the manuscript. All authors read and approved the final manuscript.

## CONFLICT OF INTEREST STATEMENT

The authors declare that they have no any competing interests (both financial and non-financial) related to this manuscript.

## Supplementary Material

Supplementary Data
