# Supplementary material for: Validity of estimated prevalence of decreased kidney function and renal replacement therapy from primary care electronic health records compared with national survey and registry data in the United Kingdom
Source: Nephrol Dial Transplant. 2017 Feb 11;32(Suppl 2):ii142–50. doi: 10.1093/ndt/gfw318 (PMC5410977; doi:10.1093/ndt/gfw318)
Supplement: Supplementary Data [file gfw318_Supp.docx]

**Supplementary data Table 1.** List of diagnosis codes indicative of renal replacement therapy in Clinical Practice Research Datalink

| Medcode | Read code | Read term |
| --- | --- | --- |
| Haemodialysis: | | |
| 2996 | 7L1A200 | Haemodialysis NEC |
| 11773 | 7L1A.11 | Dialysis for renal failure |
| 20073 | 7L1A000 | Renal dialysis |
| 20196 | 14V2.00 | H/O: renal dialysis |
| 22252 | ZV45100 | [V]Renal dialysis status |
| 28158 | TB11.00 | Kidney dialysis with complication without blame |
| 31549 | 7L1A.00 | Compensation for renal failure |
| 35921 | TA22.00 | Failure of sterile precautions during perfusion |
| 44422 | 14V2.11 | H/O: kidney dialysis |
| 46145 | ZV56011 | [V]Aftercare involving renal dialysis NOS |
| 48022 | 7L1Ay00 | Other specified compensation for renal failure |
| 54844 | U612200 | [X]Failure sterile precautions dur kidney dialys/other perf |
| 60302 | 7A60600 | Creation of graft fistula for dialysis |
| 60743 | ZV56.00 | [V]Aftercare involving intermittent dialysis |
| 64636 | 7L1Az00 | Compensation for renal failure NOS |
| 65089 | 7L1Cz00 | Placement other apparatus- compensate for renal failure NOS |
| 66714 | TB11.11 | Renal dialysis with complication without blame |
| 69266 | TA22000 | Failure of sterile precautions during kidney dialysis |
| 69427 | TA02z00 | Accid cut puncture perf h'ge - perfusion NOS |
| 69760 | ZVu3G00 | [X]Other dialysis |
| 71124 | 7L1A300 | Haemofiltration |
| 83513 | 7L1C.00 | Placement other apparatus for compensation for renal failure |
| 96184 | TA02000 | Accid cut puncture perf h'ge - kidney dialysis |
| 101756 | 7L1A011 | Thomas intravascular shunt for dialysis |
| Peritoneal dialysis: | | |
| 2994 | 7L1A100 | Peritoneal dialysis |
| 8037 | 7L1B000 | Insertion of ambulatory peritoneal dialysis catheter |
| 23773 | 7L1B100 | Removal of ambulatory peritoneal dialysis catheter |
| 30709 | 7L1C000 | Insertion of temporary peritoneal dialysis catheter |
| 30756 | 7L1A500 | Continuous ambulatory peritoneal dialysis |
| 36442 | 7L1B.11 | Placement ambulatory dialysis apparatus - compens renal fail |
| 56760 | 7L1B.00 | Placement ambulatory apparatus compensation renal failure |
| 59194 | 7L1By00 | Placement ambulatory apparatus- compensate renal failure OS |
| 64828 | 7L1A600 | Peritoneal dialysis NEC |
| 88597 | 7L1A400 | Automated peritoneal dialysis |
| Kidney transplantation: | | |
| 2997 | 7B00.00 | Transplantation of kidney |
| 5504 | 7B00z00 | Transplantation of kidney NOS |
| 5911 | ZV42000 | [V]Kidney transplanted |
| 11553 | SP08300 | Kidney transplant failure and rejection |
| 11745 | 7B00100 | Transplantation of kidney from live donor |
| 17253 | 8L50.00 | Renal transplant planned |
| 18774 | TB00111 | Renal transplant with complication without blame |
| 24361 | 7B00200 | Transplantation of kidney from cadaver |
| 26862 | 7B06300 | Exploration of renal transplant |
| 48057 | K0B5.00 | Renal tubulo-interstitial disordrs in transplant rejectn |
| 49028 | 14S2.00 | H/O: kidney recipient |
| 54990 | TB00100 | Kidney transplant with complication without blame |
| 66705 | 7B00111 | Allotransplantation of kidney from live donor |
| 70712 | SP08011 | Det.ren.func.after ren.transpl |
| 70874 | 7B00y00 | Other specified transplantation of kidney |
| 72004 | 7B01511 | Excision of rejected transplanted kidney |
| 89924 | 7B00300 | Allotransplantation of kidney from cadaver heart-beating |
| 90952 | 7B0F100 | Pre-transplantation of kidney work-up recipient |
| 93366 | 7B0F.00 | Interventions associated with transplantation of kidney |
| 94964 | 7B0F400 | Post-transplantation of kidney examination live donor |
| 96095 | 7B0F200 | Pre-transplantation of kidney work-up live donor |
| 96133 | 7B00400 | Allotransplantation kidney from cadaver heart non-beating |
| 98364 | 7B00211 | Allotransplantation of kidney from cadaver |
| 100693 | Kyu1C00 | [X]Renal tubulo-interstitial disorders/transplant rejection |

**Supplementary data Table 2.** Age-sex distribution in Clinical Practice Research Datalink and UK census 2013

| Age  (years) | Clinical Practice Research Datalink | | UK census 2013 | |
| --- | --- | --- | --- | --- |
|  | No. of people | Proportion (%) | No. of people | Proportion (%) |
| Men: |  |  |  |  |
| 25-29 | 117,261 | 4.2 | 2,171,395 | 4.9 |
| 30-34 | 120,023 | 4.3 | 2,148,903 | 4.8 |
| 35-39 | 120,256 | 4.4 | 1,975,110 | 4.4 |
| 40-44 | 136,483 | 4.9 | 2,221,431 | 5.0 |
| 45-49 | 148,763 | 5.4 | 2,310,722 | 5.2 |
| 50-54 | 144,341 | 5.2 | 2,149,309 | 4.8 |
| 55-59 | 123,154 | 4.5 | 1,854,877 | 4.2 |
| 60-64 | 108,541 | 3.9 | 1,734,599 | 3.9 |
| 65-69 | 109,100 | 4.0 | 1,697,784 | 3.8 |
| 70-74 | 81,192 | 2.9 | 1,201,622 | 2.7 |
| 75-79 | 62,543 | 2.3 | 954,347 | 2.1 |
| 80-84 | 44,307 | 1.6 | 650,871 | 1.5 |
| >=85 | 36,294 | 1.3 | 491,300 | 1.1 |
| Women: |  |  |  |  |
| 25-29 | 114,601 | 4.1 | 2,178,575 | 4.9 |
| 30-34 | 120,740 | 4.4 | 2,177,964 | 4.9 |
| 35-39 | 118,908 | 4.3 | 1,991,769 | 4.5 |
| 40-44 | 134,237 | 4.9 | 2,274,838 | 5.1 |
| 45-49 | 145,120 | 5.3 | 2,375,928 | 5.3 |
| 50-54 | 141,266 | 5.1 | 2,194,934 | 4.9 |
| 55-59 | 121,259 | 4.4 | 1,901,770 | 4.3 |
| 60-64 | 110,258 | 4.0 | 1,806,214 | 4.0 |
| 65-69 | 114,370 | 4.1 | 1,793,663 | 4.0 |
| 70-74 | 88,857 | 3.2 | 1,337,533 | 3.0 |
| 75-79 | 73,334 | 2.7 | 1,137,778 | 2.6 |
| 80-84 | 58,348 | 2.1 | 899,031 | 2.0 |
| >=85 | 68,199 | 2.5 | 967,876 | 2.2 |
| Total: | 2,761,755 | 100 | 44,600,143 | 100 |
